# Investigation of Nursing Student Satisfaction with the First Clinical Education Experience in Universities of Medical Sciences in Iran

**DOI:** 10.25122/jml-2018-0008

**Published:** 2019

**Authors:** Farzaneh Mohammad Nejad, Marziyeh Asadizaker, Shahram Baraz, Amal Saki Malehi

**Affiliations:** 1. School of Nursing and Midwifery, Ahvaz Jundishapur University of Medical Sciences, Ahvaz, Iran; 2. Nursing Care Research Center in Chronic Diseases, School of Nursing and Midwifery, Ahvaz Jundishapur University of Medical Sciences, Ahvaz, Iran; 3. Health Research Institute, Thalassemia & Hemoglobinopathy Research Center, Ahvaz Jundishapur University of Medical Sciences, Ahvaz, Iran

**Keywords:** personal satisfaction, nursing students, educational nursing research, clinical nursing research

## Abstract

Satisfaction with the experience gained in clinical settings is of great significance to nursing students and novice first-year students in particular and contributes significantly to developing basic clinical skills and competence. Accordingly, the present study aimed to examine nursing student satisfaction with the first clinical education experience.

A total of 390 second- and third-semester nursing students gaining clinical experience in general surgery, internal medicine, gynecological surgery, orthopedics, emergency, obstetrics and gynecology, ophthalmology, andrological surgery, post-CCU, and otorhinolaryngology departments participated in this analytical cross-sectional study from March to June 2017. The research instrument included the “Assessment of nursing student’s Satisfaction with First Clinical Practical Education Questionnaire: Modified Version”, consisting of three parts: demographic characteristics, 37 items, and a 10-degree visual analog scale to assess student satisfaction. Descriptive statistics were used to hypothesis test in SPSS 22.

The highest rate of student satisfaction was related to the third domain labeled “Instructor’s behavior”, and the lowest rate of student satisfaction was related to the fifth and seventh domains labeled “Emotional atmosphere and learning in the clinical setting” and “Creating appropriate learning opportunities”, respectively. The results of statistical tests suggested a statistically significant relationship between the mean satisfaction score (based on the 10-degree scale) and gender (p=0.01). However, no statistically significant relationship was observed between the mean satisfaction score and other demographic characteristics such as age, grade point average (GPA), and university type.

Student satisfaction rate varies in different domains of the questionnaire. Accordingly, it is recommended that schools of nursing and midwifery incorporate the findings of this study into their first clinical, educational experience planning and take into account the educational needs of students to bring greater satisfaction.

## Introduction

Clinical education can be defined as a learning-facilitative activity in a clinical setting aimed to make quantifiable changes in students to deliver clinical care in which the clinical instructor and students are equally involved [1]. Clinical education is the first source of learning and professional identity development in medical students [2]. As the heart of professional education, clinical education is of paramount importance as it constitutes more than half of medical students’ training time and forms the foundation of professional skill acquisition [3, 4].

Clinical experience prepares nursing students to apply clinical principles in practice to the best of their theoretical knowledge [5]. The major success of nursing education programs is arguably related to students’ practical clinical experience [6]. The first clinical experience is of particular significance as it is the time when an individual accepts nursing as the profession he/she has chosen [7]. However, evidence suggests that the first clinical experience is associated with maximum tension and anxiety for students [8], which can be accounted for lack of interest and motivation on their part [9]. The anxiety experienced by nursing students is rooted in their assumed lack of adequate expertise and knowledge to deliver patient care, which results in the dissatisfaction of the majority of nursing students with their clinical education programs [5]. Since “nursing principles and techniques” is a basic course for learning clinical skills, the level of learning and effective factors in its teaching and learning can be of particular significance in clinical skill acquisition by nursing experts [10]. Nursing students are on the verge of adulthood, have just left the school environment and entered the unknown and complex clinical settings that require interaction despite their human communication intricacies. Such an experience can pose various problems to them in different ways [11].

Students almost always regard the first day of clinical learning activities in a new clinical setting as a stressful event. The first encounter either enhances learning independence in them or results in more dependence on instructors due to fear [12]. Descriptive information on the status quo and student satisfaction with provided services are necessary to bring constructive changes in any educational system. This information can be used to improve strengths, overcome weaknesses, and gain maximum student satisfaction [13]. According to educational psychologists, student satisfaction promotes self-confidence, resulting in skill development and knowledge acquisition [14]. Since students represent an essential element and a primary recipient of higher education, their views are considered essential in university quality monitoring across the world [15] and identifying clinical education problems [16].

Studies on the condition of clinical education are indicative of student dissatisfaction [17, 18], which may lead to a lack of clinical proficiency [19]. Studies attach great significance to satisfaction with the clinical education condition, which may vary with time and place [17, 18]. The study of student satisfaction in academic environments can contribute significantly to academic course quality improvement and adaptation to student needs [20]. Numerous studies have delved into issues related to student satisfaction and have come to the conclusion that, under similar conditions for other factors, students with higher levels of satisfaction are more likely to devote greater effort (e.g., regular attendance and more involvement in classroom activities), continue education, and graduate successfully [21, 22].

Given the above findings on the abundance of significant problems in the process of gaining experience by nursing students in clinical fields, student dissatisfaction with clinical education, the first clinical experience being regarded as the professional foundation of nursing education, and the limited number of studies evaluating nursing student satisfaction with the first clinical learning experience process using a valid and reliable instrument, the present study aimed to examine nursing student satisfaction with the first clinical experience.

## Materials and Methods

This analytical cross-sectional study was conducted from March to June 2017 on 390 second- and third-semester nursing students studying in 15 universities of medical sciences (Ahvaz, Iran, Shahid Beheshti, Shiraz, Golestan, Lorestan, Kermanshah, Ilam, Hamadan, Kashan, Qazvin, Dezful, Shahr-e Kord, Arak, and Jahrom) who had completed the principles and clinical skills of a nursing internship program. The samples were selected using stratified random sampling. To this end, all the specified universities of medical sciences were listed under types 1, 2, and 3. The number of nursing students entering each year in each university type was also calculated to increase data collection accuracy, the frequency of universities and student population in terms of type. Then, 390 students were included in 23 to 28-member groups based on the university type classification and the number of students covered by each university type to determine the number of universities required per type.

After a brief in-person description of the research objectives and different parts of the questionnaire, the questionnaires were administered to eligible students. Data were collected using the “Assessment of nursing student’s Satisfaction with First Clinical Practical Education Questionnaire: Modified Version”. This modified questionnaire was developed, psychometrically assessed, and verified in terms of validity and reliability by Asadizaker et al. It comprises three main parts: the first part is related to students’ personal characteristics including age, gender, high school diploma discipline, high school diploma GPA, university and university type, ward, and hospital. The second part consists of 37 items designed to evaluate nursing student satisfaction with the first clinical education experience and classified in 7 domains as follows: 1- instructor performance consisting of 9 items, 2- Coherence of the curriculum consisting of 7 items, 3- Instructor’s behavior consisting of 4 items, 4- Attention to students’ feelings and perceptions consisting of 7 items, 5- Emotional atmosphere and learning in the clinical setting consisting of 4 items, 6- creating a favorable condition to enter the profession consisting of 3 items, and 7- Creating appropriate learning opportunities consisting of 3 items. Items were responded on a 5-point Likert scale (‘completely dissatisfied’ to ‘completely satisfied’) and scored as follows: completely satisfied = 5, satisfied = 4, relatively satisfied = 3, dissatisfied = 2, and completely dissatisfied = 1. The third part examined student satisfaction with the overall “principles and clinical skills of nursing” internship program using a 10-degree visual analog scale from 1 to 10 (with 1 and 10 denoting the minimum and maximum satisfaction levels, respectively). After the questionnaires were filled out and collected, data were entered into SPSS 22 and analyzed using descriptive statistical tests.

### Ethical Consideration

The present study was conducted after obtaining permission from the Vice-Chancellor of research of Ahvaz Jundishapur University of Medical Sciences, Ahvaz, Iran with the code of ethics IR.AJUMS.REC.1396.62. After explaining the research goals, the freedom to participate in the study, and confidentiality of the information, the researcher invited university professors and students interested in participating in the study.

### Findings

This study was conducted on 390 second- and third-semester nursing students. The response rate and the student age range were 100% and 19-23 years, respectively. The majority (68.2%) of participants were female. The high school diploma discipline of the majority (96.41%) of students was Empirical Sciences. Second- and third-semester students represented 81.53 and 18.47% of students. Data were collected from 10 clinical departments with a nursing student distribution as follows: general surgery (50.76%), internal medicine (11.53%), gynecological surgery (11.02%), orthopedics (8.2%), emergency (6.92%), obstetrics and gynecology (3.84%), ophthalmology (3.07%), andrological surgery (2.05%), post-CCU (1.79%), and otorhinolaryngology (0.76%) (Table 1).

**Table 1:** Demographic Characteristics

Individual Characteristics		Frequency	Percentage
Age	Under 20 years	202	51.7
	20-22 years	144	36.92
	Above 22 years	44	11.28
Gender	Female	266	68.20
	Male	124	31.79
High School Diploma GPA	Less than 17	97	24.87
	17-19	201	51.53
	Over 19	92	23.58
High School Diploma Discipline	Empirical Sciences	376	96.41
	Mathematics	12	3.07
	Humanities	2	0.51
Academic Semester	Second semester	318	81.53
	Third semester	72	18.46
University	Ahvaz	26	6.66
	Iran	26	6.66
	Shahid Beheshti	27	6.92
	Shiraz	26	6.66
	Golestan	26	6.66
	Lorestan	26	6.66
	Kermanshah	26	6.66
	Ilam	28	7.1
	Hamadan	26	6.66
	Kashan	26	6.66
	Qazvin	26	6.66
	Dezful	26	6.66
	Shahr-e Kord	23	5.89
	Arak	26	6.66
	Jahrom	26	6.66
University Type	Type 1	105	26.92
	Type 2	182	46.66
	Type 3	103	26.41
Clinical Ward	General surgery	198	50.76
	Gynecological surgery	43	11.02
	Andrological surgery	8	2.05
	Internal medicine	45	11.53
	Obstetrics and gynecology	15	3.84
	Orthopedics	32	8.2
	Emergency	27	6.92
	Ophthalmology	12	3.07
	Otorhinolaryngology	3	0.76
	Post-CCU	7	1.79

Satisfaction levels for individual items were examined to determine the highest and lowest student satisfaction rate with each domain. The highest student satisfaction rate was related to the third domain labeled “Instructor’s behavior”, and the lowest student satisfaction rate was related to the fifth and seventh domains labeled “Emotional atmosphere and learning in the clinical setting” and “Creating appropriate learning opportunities”, respectively (Table 2).

**Table 2:** The student satisfaction rate with different domains

Domain	Items	Completely satisfied	satisfied	relatively satisfied	dissatisfied	Completely dissatisfied
Instructor’s performance	The instructor provides learning opportunities to observe and engage students.	33.2	31.6	21.9	8.4	4.8
	The instructor directs and guides students during the implementation of nursing care provided for patients.	34.9	38.0	16.3	9.7	1.0
	The instructor has enough capability to perform clinical nursing skills.	39.5	30.5	22.4	6.1	1.0
	The instructor teaches students skills to effectively communicate with the patient and family.	29.1	36.0	23.2	9.4	2.3
	The instructor establishes continuous and dynamic engagement with the nurses.	36.0	40.8	16.8	5.1	1.0
	The instructor observes the educational discipline and rules such as timely attending the department, not quitting the internship, etc.	39.3	33.4	17.6	6.1	3.6
	The instructor establishes continuous and dynamic interactions with the head nurse.	36.0	39.8	19.9	2.8	1.5
	The instructor’s interest in the nursing profession enhances the students’ levels of satisfaction and desire.	34.9	37.2	19.1	5.9	2.8
	The instructor introduces the students to the staff on the first day of the internship.	23.5	33.7	20.9	14.3	7.7
Coherence of the curriculum	The method and instruments of internship assessment are specified by the instructor.	24.0	41.6	25.0	5.9	3.6
	On the first day, the written schedule of the entire unit of internship is given to the students by the instructor.	28.8	31.4	28.3	8.7	2.8
	The performance of each student is evaluated by the instructor using the logbook.	35.2	32.1	25.8	4.3	2.6
	The lesson plan is provided to the students on a daily basis, in writing and verbally.	15.3	36.2	30.4	14.0	4.1
	Faculty members address the students’ needs and problems by monitoring the clinical education process.	17.6	32.9	29.6	13.5	6.4
	Clinical education is implemented in accordance with the goals and schedule of the internship.	26.8	38.0	24.0	8.9	2.3
	Head nurse or ward officials are aware of the students’ daily schedules.	22.2	38.0	24.5	11.5	3.8
Instructor’s behavior	The instructor treats students with a high degree of patience and calm during the internship.	50.0	30.1	14.3	4.3	1.3
	The instructor’s behavior and performance is a good model for students.	37.0	34.5	21.2	6.6	1.0
	The instructor is enough capable of providing accurate and correct answers to students’ academic questions.	35.5	36.5	25.5	1.5	1.0
	The instructor has decent approval rating among the students.	34.9	29.3	28.8	5.4	1.5
Attention to students’ feelings and perceptions	The instructor gives verbal and non-verbal feedback to students about the care provided by them.	22.2	24.1	21.9	9.9	1.8
	The mental and emotional atmosphere of the clinical learning setting is positive.	23.2	35.2	24.5	12.0	5.0
	Students learn to overcome the stress caused by the first clinical experience with the help of the instructor.	31.9	35.7	20.4	8.2	3.8
	The content of clinical education is designed from simple to complex.	25.8	38.0	28.3	5.6	2.3
	I feel relaxed with my instructor.	31.4	34.9	23.7	8.7	4.1
	There is a harmony between the instructor’s expectations and my ability.	23.5	40.1	23.7	8.7	4.1
	The instructor supports students during the internship.	35.5	23.4	20.4	9.2	1.5
Emotional atmosphere and learning in the clinical setting	The nurses of the ward were friendly to the students in their first contact.	19.6	30.6	29.3	14.3	6.1
	Nurses cooperate with the instructor while training students.	17.9	31.9	26.8	12.5	11.0
	Nurses and instructors provide the students with required facilities available in the department, such as the blood pressure monitor, educational pamphlets, etc.	21.2	32.4	29.3	12.2	4.8
	The atmosphere of learning settings creates a sense of being a nurse in the students.	32.9	32.4	24.0	8.2	2.6
creating a favorable condition to enter the profession	The internship provided a good opportunity for students to predict future job responsibilities.	34.7	39.8	20.4	3.8	1.3
	This internship is considered the first positive clinical experience.	32.1	40.6	21.4	4.3	1.3
	Students feel satisfied at the end of the internship.	31.1	37.5	20.7	7.7	3.1
Creating appropriate learning opportunities	There is a good balance between the number of students and instructors, so that the instructor can provide the student with appropriate education.	23.2	32.1	19.9	11.5	13.3
	The instructor and the students will have the opportunity to learn and practice techniques during the internship.	24.2	36.2	25.5	11.2	2.8
	Students are sufficiently familiarized so that they are well prepared for the clinical setting.	26.3	31.9	28.6	10.7	2.6

The score range of each domain was calculated given the number of items in each domain and the Likert scale scoring system for each item where scores 5, 4, 3, 2, and 1 denoted completely satisfied, satisfied, relatively satisfied, dissatisfied, and completely dissatisfied, respectively. The score ranges of domains one to seven were as follows: 9-45, 7-35, 4-20, 7-35, 4-20, 3-15, and 3-15, respectively.

The mean student satisfaction scores (based on the Likert scale) were then calculated in terms of demographic characteristics for each domain (Table 3).

**Table 3:** The mean student satisfaction scores (based on the Likert scale) in terms of students’ demographic characteristics for the instrument domains

Demographic Characteristics		Mean Satisfaction Score						
		1^st^ domain	2^nd^ domain	3^rd^ domain	4^th^ domain	5^th^ domain	6^th^ domain	7^th^ domain
Gender	Male	43.54	25.92	15.86	26.56	14.74	11.90	10.58
	Female	35.44	25.62	16.31	26.36	13.86	11.48	10.58
Age	Under 20 years	34.75	25.83	16.29	26.33	13.65	11.86	10.81
	20-22 years	36.12	25.83	16.25	27.44	15.05	11.99	10.52
	Above 22 years	34.79	25.86	15.72	25.40	14.18	11.86	11.43
GPA	Less than 17	34.17	25.67	15.86	26.67	14.62	11.82	11.19
	17-19	35.78	25.63	16.45	26.48	13.89	11.89	10.61
	Over 19	35.26	25.23	16.03	26.29	14.23	11.86	10.76
University Type	Type 1	31.42	24.63	15.56	24.08	13.24	11.17	9.99
	Type 2	37.64	24.40	16.81	27.85	14.20	12.12	11.12
	Type 3	34.57	25.62	15.67	26.33	14.93	12.10	10.94

The overall mean score of student satisfaction with the overall principles and clinical skills of the nursing internship was calculated to be 7.48 using the 10-degree visual analog scale. The Analysis of Variance (ANOVA) results suggested a statistically significant relationship between the mean satisfaction score (based on the 10-degree scale) and gender (p=0.01). However, no statistically significant relationship was observed between the mean satisfaction score and other demographic characteristics such as age, GPA, and university type (Table 4).

**Table 4:** The overall mean satisfaction score in terms of student demographic characteristics (based on the 10-degree visual analog scale)

Demographic Characteristics		Mean Satisfaction Score
Gender	Male	7.32
	Female	7.56
Age	Under 20 years	7.45
	20-22 years	7.56
	Above 22 years	7.45
GPA	Less than 17	7.58
	17-19	7.38
	Over 19	7.60
University Type	Type 1	7.36
	Type 2	7.62
	Type 3	7.39

## Discussion

The present study aimed to investigate nursing student satisfaction with the first clinical education experience in Iranian universities of medical sciences. The study of student views on the current educational system status can undoubtedly increase their satisfaction and shed light on the existing problems in the current educational system and the reasons behind lack of interconnection between students and the educational system [23].

The first domain examines student satisfaction with instructor performance in providing proper clinical education and communication skills training, establishing consistent interaction with other educational elements, and observing educational principles.

The second domain provides accurate information to students on the methods of educational delivery, and coherence between the nursing schools and clinical departments in the first clinical experience of students. Reimer-Kirkham et al. [24] and Yang et al. [25] support the necessity of paying attention to coherence in the clinical education structure to improve student learning through clarification of educational goals, proper design of learning activities, and clinical education innovations proportional to educational programs.

The third domain investigates student satisfaction with the proper behavior of instructors toward students, as well as their proficiency and place as role models. According to Tavakoli’s research, individual personality traits, educational skills, and interpersonal relationships play a decisive role in the efficacy of instruction [26].

The fourth domain is associated with student satisfaction with the way the instructor helps them overcome their stress caused by the first clinical encounter, achieve peace of mind, meet his/her reasonable expectations, and receive support and feedback. Similarly, Sharifnouri indicated that first-year nursing students undergo considerable stress mostly associated with clinical elements [27]. Other studies also indicate the significance of the supportive, leading, encouraging, facilitative, and guiding role of clinical instructors as well as their clinical competence, good manners, and diligence in the quality of clinical education [28, 29].

The fifth domain is related to student satisfaction with the facilities and equipment of the clinical setting and student-professional nursing staff interactions. Nazari et al. pointed out that the clinical setting features are of profound significance and the consistency of therapeutic care goals of clinical departments with instructors’ educational goals can positively affect clinical education [30]. Compatibility between education and the clinical setting in the nursing profession necessitates cooperation between instructors of nursing schools and professional nursing staff, the realization of which finally ensuring the progress of the nursing profession [31].

The sixth domain is associated with student satisfaction with the provision of a proper opportunity at the conclusion of the internship program to understand vocational nursing responsibilities. Elliot & Shin maintain that student satisfaction assessment allows universities to change their programs in accordance with student needs and to develop a system that continually monitors such programs [32].

The seventh and final domain investigates student satisfaction with the instructor availability and creation of learning opportunities. Pazargadi stresses the necessity of instructor availability and attendance in the clinical department as well as his/her cooperation in tasks and with students [33]. Learning clinical skills requires gaining clinical experience as a student and practicing skills through observation, collaboration, following clinical procedures, drawing inferences, and managing patients under the instructor’s supervision. This is because the goal of clinical education is to provide opportunities that enable students to link their theoretical knowledge with practical realities [34].

## Conclusion

The results revealed that the highest student satisfaction rate was related to the “Instructor’s behavior”, and the lowest student satisfaction rate was related to the “Emotional atmosphere and learning in the clinical setting” and “Creating appropriate learning opportunities.” The results of statistical tests suggested a statistically significant relationship between the mean satisfaction score (based on the 10-degree scale) and gender.

## Acknowledgment

This article is derived from a nursing master’s thesis of Farzaneh Mohammadnejad, which was conducted with support from Ahvaz Jundishapur University of Medical Science The researcher hereby would like to express her gratitude to all esteemed students who made this study possible.

## Conflict of Interest

The authors confirm that there are no conflicts of interest.
